# ReViTA-Unet: An Enhanced Semantic Segmentation Model for Automated Morphometric Analysis of *Macrobrachium rosenbergii*

**DOI:** 10.3390/s26144570

**Published:** 2026-07-19

**Authors:** Dawei Sun, Qi Chen, Guanghui Yu, Xinran Han, Chen Li, Chengquan Zhou, Hongbao Ye

**Affiliations:** 1 Institute of Agricultural Equipment, Zhejiang Academy of Agricultural Sciences, Hangzhou 310021, China; dzs0015@zju.edu.cn (D.S.); chenqi0011@foxmail.com (Q.C.); ghuiy@cau.edu.cn (G.Y.); lichen@zaas.ac.cn (C.L.); 2 Key Laboratory of Agricultural Equipment for Hilly and Mountainous Areas in Southeastern China (Co-Construction by Ministry and Province), Ministry of Agriculture and Rural Affairs, Hangzhou 310021, China; 3 East China Sea Fisheries Research Institute, Chinese Academy of Fishery Sciences, Shanghai 200090, China; 19863823932@163.com

**Keywords:** prawn, *Macrobrachium rosenbergii*, deep learning, vision transformer, precision agriculture

## Abstract

**Highlights:**

**What are the main findings?**

- ReViTA-UNet synergistically integrates Res-CNN, Vision Transformer, and ASPP with U-Net, achieving superior segmentation of complex prawn morphology (Dice = 97.7%, mIoU = 96.7%).
- A geometric topological repair algorithm was developed and incorporated, further enhancing measurement accuracy and achieving a body length MAPE of 1.83% (R^2^ = 0.987).

**What are the implications of the main findings?**

- The automated, non-contact measurement system may decrease handling stress, reduce labor, and enable high-throughput phenotyping.
- The framework exhibits potential for precision aquaculture management, with future commercial deployment subject to further validation.

**Abstract:**

Accurate morphometric analysis of *Macrobrachium rosenbergii* is essential for selective breeding, growth monitoring, and precision aquaculture, yet conventional manual measurements are labor-intensive, time-consuming, and prone to operator variability. This study presents ReViTA-UNet, an automated, non-contact morphometric analysis framework based on an enhanced semantic segmentation network coupled with a geometric topology refinement algorithm to accurately extract multiple morphological traits. The proposed framework integrates complementary feature extraction to improve segmentation of elongated anatomical structures and complex body boundaries. A complete automated measurement system was subsequently developed to convert segmented images into biologically meaningful morphometric parameters. The results demonstrated that ReViTA-UNet achieved a Dice coefficient of 97.7%, a mean Intersection over Union (mIoU) of 96.7%, a precision of 98.3%, and a recall of 98.4%, outperforming eight representative semantic segmentation models. The automated measurement system achieved a mean absolute percentage error of 1.83% for body length, with strong agreement with manual measurements (R^2^ = 0.987), while maintaining high accuracy for other major morphometric traits. These results indicate that the proposed framework provides an accurate and efficient solution for automated prawn phenotyping under controlled imaging conditions. It establishes a practical foundation for future intelligent aquaculture applications following validation under commercial farming environments.

## 1. Introduction

*Macrobrachium rosenbergii*, also known as the giant freshwater prawn, is an intensively cultured freshwater crustacean species in global aquaculture, particularly throughout tropical and subtropical regions, especially in Asia. It provides an important source of dietary protein and economic livelihood for millions of small-scale farmers and commercial enterprises. Its high market value and adaptability to diverse culture systems underscore its importance in sustainable aquaculture development [1]. However, continuous improvement and large-scale aquaculture of M. rosenbergii require the coordinated optimization of multiple components, including hatchery management, selective breeding, water quality management, feed provision, disease prevention, and growth monitoring. These efforts, however, remain constrained by conventional methods for measuring key morphometric traits, including body length, body width, and carapace length [2]. Accurate morphometric assessment is therefore essential for breeding programs, aquaculture management, and production-related decision-making.

Nevertheless, current husbandry practice still relies heavily on manual and invasive sampling, in which individual prawns are captured, often anesthetized, physically measured, and visually assessed. Such procedures are labor-intensive, inefficient, and inherently vulnerable to errors owing to variations in experience among personnel [3]. The absence of standardized protocols further compromises representativeness, particularly in high-density and large-scale farming systems. Moreover, M. rosenbergii is highly stress-sensitive, which can trigger autotomy (limb loss), suppress feeding, increase aggression, and increase mortality rates, thereby directly confounding growth and welfare data [4]. Repeated handling has also been shown to alter physiological indices and distort genetic parameter estimates, ultimately compromising selection accuracy [5]. Consequently, traditional manual measurement methods are inadequate to meet the demands for precision and intelligent management in modern aquaculture [3]. Therefore, there is an urgent need to develop noninvasive and rapid measurement techniques.

The application of machine vision technology to achieve automated non-contact measurement of prawns has emerged as an important research direction in intelligent aquaculture. Early studies predominantly employed traditional image-processing methods, such as threshold segmentation and edge detection. Harbitz established a log-linear model between length and pixel area, utilizing image intensity threshold segmentation to estimate the body length of northern shrimp [6]. Pan et al. [7] applied a histogram bimodal threshold method to segment shrimp bodies. They subsequently used morphological features to predict body weight. To address the issue of shrimp contact in high-density scenarios, Liu et al. [8] proposed a segmentation technique based on an improved pruning algorithm using the watershed method. However, under practical farming environments, M. rosenbergii exhibits distinct biological characteristics. Its body parts, such as the long rostrum, slender pereiopods, and telson, exhibit distinct morphologies. These features reduce the robustness of traditional algorithms during feature extraction and hinder high-precision edge segmentation.

In recent years, deep-learning technologies represented by Convolutional Neural Networks (CNNs) have achieved breakthroughs in semantic segmentation. Mainstream approaches to aquatic target detection and segmentation generally fall into two categories: instance segmentation algorithms based on one-stage object detection and end-to-end semantic segmentation architectures. For example, Ran et al. [9] employed an improved YOLOv8 algorithm to achieve high-precision instance segmentation of Prawn and combined it with statistical methods to develop a model for identifying abnormal individual growth. Abiam et al. [10] developed an automated analysis system Imashrimp that utilizes a transformer-based pose estimation module to predict 23 skeletal key points in shrimp, thereby enhancing the dimensionality of biomass assessment. Hong et al. [11] used Mask R-CNN for shrimp segmentation and counting, and reported an accuracy of 97.48%. Lai et al. [12] applied the YOLOv4-tiny algorithm to detect shrimp bodies, followed by cropping and segmentation using the Otsu algorithm, and obtained the pixel length and width of the shrimp through minimum rectangular fitting. A critical step in translating pixel-level features into biological phenotypes is accurate calibration between pixel dimensions and true physical length. Various optical 3D vision systems have been proposed to estimate underwater fish length. Structured light systems project specific light patterns to acquire high-density point cloud data of biological surfaces [13], whereas stereo vision systems leverage multi-view disparity principles combined with refraction compensation algorithms [14] to address the lack of depth perception in monocular vision and enable accurate, non-contact reconstruction of spatial postures and multi-dimensional morphological parameters of aquatic organisms, such as shrimp and fish. Vision Transformers (ViTs) have recently been adopted for semantic segmentation, leveraging self-attention to model long-range spatial dependencies across the entire image. Representative works include SETR [15], which first adapted the pure Transformer encoder for segmentation, and Swin Transformer [16], which introduced hierarchical representations with shifted windows for efficient multi-scale processing. These developments motivate our adoption of ViT architecture to address the unique morphological challenges of prawn segmentation.

In this study, we present and design an enhanced semantic segmentation model named ReViTA-UNet for high-precision segmentation and non-contact automated measurement of M. rosenbergii. By synergistically incorporating multiple validated modules in a principled manner, including Residual convolution (Res-CNN), Vision Transformer (ViT), and Atrous Spatial Pyramid Pooling (ASPP), the model substantially improves the extraction of fine-scale prawn features from images in a rapid, noninvasive manner. We further developed a supporting automated measurement system integrated with geometric topology restoration and parameter calculation functions, thereby enabling automated conversion of raw images into biologically quantifiable metrics. Therefore, the proposed automated measurement system provides technical support for intelligent aquaculture management. The innovations of this study are summarized as follows:

1. A task-oriented semantic segmentation framework, ReViTA-UNet, was developed for accurate segmentation of giant freshwater prawns. The proposed framework effectively integrates residual convolution, Vision Transformer, atrous spatial pyramid pooling (ASPP), and attention-guided upsampling to address the elongated appendages, scale variation, and complex anatomical boundaries characteristic of *Macrobrachium rosenbergii*.
2. A geometric topology refinement algorithm was developed to improve automated morphometric analysis. By combining skeleton breakpoint reconnection, projection-based compensation, constrained Voronoi medial-axis extraction, and curve fitting, the proposed approach enhances skeleton continuity and measurement robustness, particularly for curved and slender anatomical structures.
3. An end-to-end automated morphometric analysis system was established. The developed system integrates semantic segmentation, geometric topology reconstruction, and automated trait extraction into a unified workflow, enabling rapid, non-contact, and reproducible measurement of multiple prawn morphometric traits under controlled imaging conditions. This framework provides a practical foundation for future intelligent phenotyping and precision aquaculture applications.

The remainder of the manuscript is organized into three main sections. Section 2 details the dataset development, network architecture of the proposed ReViTA-UNet model, and construction of an automatic real-time measurement system. Section 3 comprehensively evaluates the model and presents comparisons of optimization strategies, ablation experiments at varying sparsification ratios, and performance benchmarks against contemporary state-of-the-art models, followed by a discussion of the findings. Section 4 concludes the manuscript.

## 2. Materials and Methods

### 2.1. Dataset

#### 2.1.1. Specimen and Image Acquisition System

**Figure 1:**
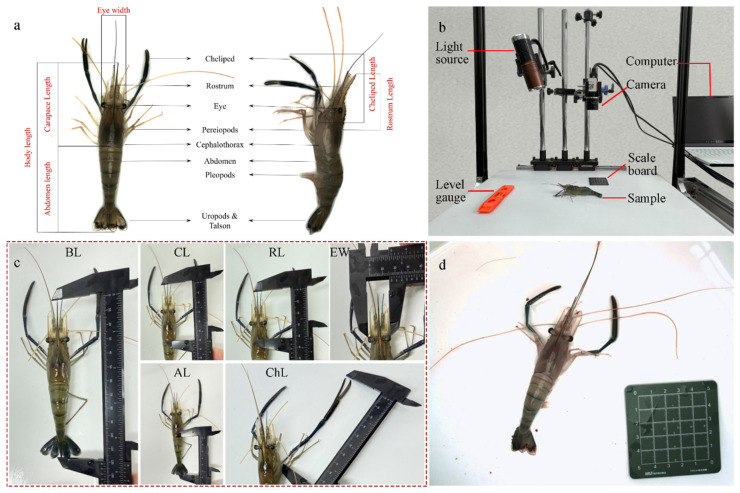
Data acquisition. (**a**) Morphology of *Macrobrachium rosenbergii* and key features in this study; (**b**) system settings for image acquisition. (**c**) Manual measurement, body length (BL), carapace length (CL), abdomen length (AL), rostrum length (RL), cheliped length (ChL), and eye width (EW); (**d**) sample image.

Images were acquired under a controlled laboratory environment. The prawn samples were sourced from multiple vendors. The dataset included samples collected over 2 years, and a total of 682 prawns were obtained for image acquisition. The prawns weighed 20.5 ± 3.71 g, capturing variation in specimen size. The parameters selected in this study included body length, carapace length, abdominal length, rostrum length, cheliped length, and eye width, as illustrated in Figure 1a. Prior to image acquisition, key morphometric data were obtained through manual measurements of all prawn specimens. The image acquisition process was conducted using a custom-developed image acquisition platform. The image acquisition system included an industrial RGB camera (HE020E1GC, Huayong Tech, Shenzhen, China) for high-resolution images and a uniform light source (5000 K color temperature) to provide consistent white-light illumination, mounted on a rigid frame. A dedicated computer captured the images and streamlined the analytical workflow. The system setting was shown in Figure 1b. To compare the performance of model results, detailed each morphological trait was manually measured by capillary, as shown in Figure 1c.

#### 2.1.2. Data Acquisition

To ensure precise and consistent image capture, prawn images were acquired using a top-down camera setup. An industrial RGB camera was mounted vertically at a fixed height of 32.5 cm above the measurement platform (Figure 1b). This position provided an optimal field of view for imaging the region of interest, balancing feature resolution and ensuring capture of both the full prawn profile and key morphological details. For ongoing calibration, a reference scale board was included within the field of view during every imaging session to ensure calibration accuracy and measurement consistency across specimens as shown in the sample image (Figure 1d). The images were recorded at a resolution of 1280 × 1024 pixels, ensuring preservation of fine anatomical details.

#### 2.1.3. Data Preprocessing

**Figure 2:**
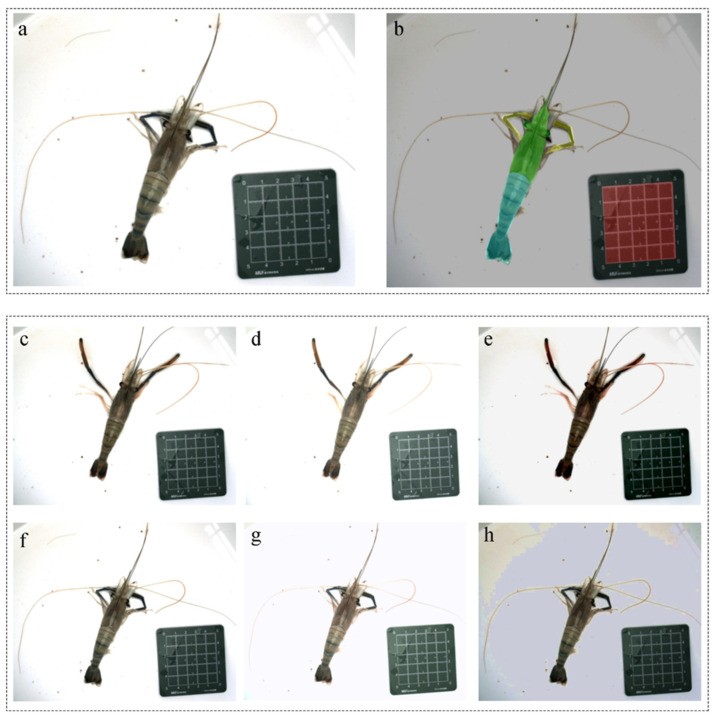
Data preprocessing for accurate prawn measurement. (**a**) original image; (**b**) Manual labeling using LabelMe tool; (**c**,**f**) sample original image; (**d**) strong backlight effect; (**e**) simulated random shadow effect; (**g**) sharpening effect; (**h**) simulated fog effect.

A total of 682 *Macrobrachium rosenbergii* specimens were collected, and manual morphometric measurements were recorded for each individual prior to image acquisition. For semantic segmentation, the LabelMe annotation tool was used to generate pixel-level annotations. Owing to the labor-intensive nature of semantic annotation and the need to ensure consistent labeling across multiple anatomical regions (carapace, rostrum, abdomen, and chelipeds), a representative subset of 170 images was manually annotated. Rather than single-target detection, a multipart semantic segmentation strategy was employed, dividing the prawn into functional regions. A standard calibration block was annotated in each image to facilitate subsequent length measurements of different body sections. This detailed, category-specific, pixel-wise labeling provides a precise data foundation for later extraction of the prawn’s central axis or skeletal key points, enabling the fitting of a true body length curve. Annotated segmentation images are shown in Figure 2.

The annotated dataset was randomly divided into training and validation sets at a ratio of 8:2, resulting in 136 training images and 34 validation images. Data augmentation techniques were applied to enhance the generalization capability and improve robustness of the deep learning model against varying imaging conditions. To prevent data leakage, data augmentation was applied exclusively to the training set. All five variants of a given training image were assigned to the training set, while no augmentation was applied to the validation set. This ensures that the validation set remains completely independent and reflects the model’s ability to generalize to unseen specimens. After data augmentation, the effective training set comprised 680 images (136 original × 5 augmentations). The validation set was held completely independent and used exclusively for final performance evaluation. Further, to provide a completely independent evaluation of the measurement pipeline, a test set of 100 images was randomly selected from the remaining 512 unannotated specimens which were never used for segmentation training or validation, ensuring unbiased assessment of measurement accuracy. Consequently, the final datasets used in this study consisted of 680 training images, 34 validation images, and 100 independent test images. The data preprocessing workflow, annotation examples, and representative augmentation results are illustrated in Figure 2.

### 2.2. Model Construction

#### 2.2.1. The Construction of ReViTA-UNet

As a classic symmetrical fully convolutional neural network, the core value of U-Net lies in its distinctive encoder–decoder architecture [17]. The encoder module is designed to extract global contextual information and perform feature downscaling, whereas the decoder module integrates feature maps from multiple receptive fields through concatenation operations. This approach compensates for spatial information loss during upsampling and substantially improves the model’s localization accuracy for complex target objects.

**Figure 3:**
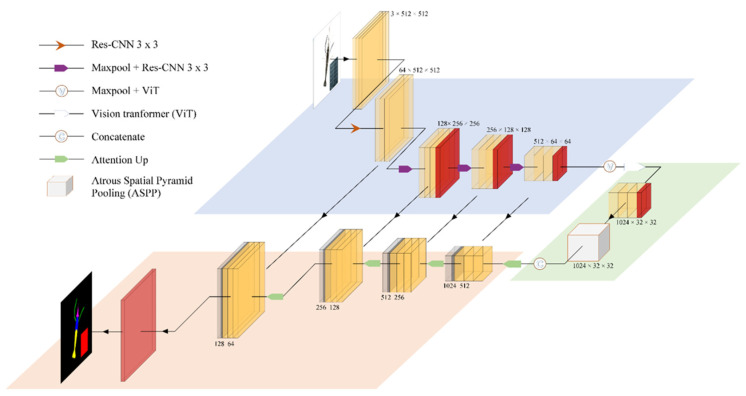
The structure of ReViTA-Unet.

This study presents an enhanced ReViTA-UNet-based segmentation model for prawns. Its core architecture follows the symmetric encoder–decoder paradigm as illustrated in the network structure diagram. In the encoder, conventional convolutional blocks are replaced with residual modules with identity mapping to alleviate gradient vanishing and improve propagation of fine-grained textural features. To capture the long-range spatial dependencies between prawn appendages during feature compression, a ViT block was embedded in the deepest encoding unit. Leveraging the self-attention mechanism enhances global contextual modeling and significantly improves recognition accuracy for the logical relationships between the claws and the body under complex postures. An ASPP module was then integrated into the bottleneck layer. Through parallel multi-scale dilated convolutions, the receptive field is further expanded to accommodate large morphological size variations in the prawn limbs. Finally, in the decoder path, attention-gated feature fusion is applied to refine spatial feature weighting and improve boundary localization accuracy. The structure of Re ReViTA-Unet is shown in Figure 3.

#### 2.2.2. Res-CNN Residual Convolutional Module

**Figure 4:**
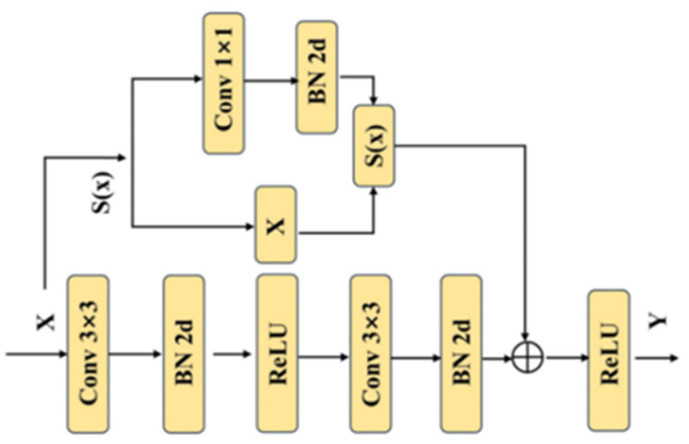
Res-CNN residual convolution block used in this study.

To prevent the loss of deep features as the network depth increases, and to accurately capture the complex morphological details of prawns while preserving their feature information, this study designed the Res-CNN module as the core computational unit of the ReViTA-UNet encoder. The architecture of the module is shown in Figure 4.

The core of the Res-CNN is the decomposition of the target mapping H(x) into the sum of the linear identity mapping and non-linear residual mapping. The main path consists of two sequential convolutional operators, followed by batch normalization and a ReLU activation function. Its nonlinear transformation, denoted F(X), is expressed as follows:

(1) $$F\left(X\right)={BN}_{2}({Con}_{3\times 3}(\sigma \left({BN}_{1}\left({Con}_{3\times 3}\left(X\right)\right)\right)))$$

where *Con*_3×3_ denotes a convolutional operation with a 3 × 3 kernel size, *BN* refers to batch normalization, which stabilizes the distribution of neuron activations across the network, and *σ* represents the ReLU activation function, enhancing the model’s nonlinear expressive capability.

To enable element-wise addition between the main path and input features, their dimensional spaces must be consistent. In this study, an adaptive shortcut mechanism *S*(*x*) was designed. When the input and output channel dimensions matched, direct identity mapping was applied. When the dimensions were incompatible, a 1 × 1 convolution was used to align them. The formula used is as follows:

(2) Sx=x,BNCon1×1x,if Cin=Coutif Cin≠Cout

(3) Y=σ(F(X)⊕S(x))

where *BN* stands for batch normalization and ⊕ denotes element-wise addition. The Res-CNN module establishes a “highway for information flow” through residual connections, offering distinct advantages when applied to the segmentation task of prawn. Residual learning allows gradients to propagate directly through skip connections, bypassing weight layers, which enhances the sensitivity of the network to small-area, high-edge features such as the slender claws of the prawn. Furthermore, the stacked dual-layer 3 × 3 convolutional blocks maintain parameter efficiency while effectively modeling complex textures near the limb joint regions. This design ensures both anatomical coherence and completeness of the segmentation results.

#### 2.2.3. Vision Transformer Deep Global Attention Modeling

**Figure 5:**
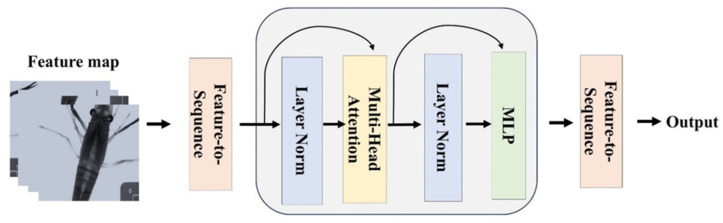
Deep global modeling module based on Vision Transformer.

In traditional convolutional neural networks, models often struggle to effectively capture long-range spatial dependencies in deeper layers, owing to the inherent limitations of receptive fields. To address the complex limb topology of prawns, this study embedded a ViT module after the fourth downsampling layer, aiming to fully exploit long-range spatial dependencies in a high-dimensional semantic space. By leveraging the global self-attention mechanism of a vision transformer, the model achieved a paradigm shift from localized feature extraction constrained by limited receptive fields to extensive contextual semantic correlations [18]. The diagram of this module is provided in Figure 5.

In the fourth layer, the input features first underwent spatial compression via residual convolution. To meet the input requirements of the transformer, the module performs tensor flattening and transposition operations and maps the spatial dimensions to the sequence dimension. The formula used is as follows:

(4) $$X_{seq}=\mathrm{Reshape}\left(X_{local}\right)\in R^{N\times C},N=H\times W$$

Here, *X_local_* represents the output from the previous layer, with the parameters in this study being *H* = *W* = 32, *N* = 1024, where *N* denotes the sequence length mapped from the spatial positions. Through this transformation, pixel points that are spatially distant in the image, such as the tip of the prawn claw and tail, achieve equidistant mapping in the sequence dimension.

Compared to the traditional ViT, this approach eliminates the need to add positional encodings. By leveraging the translation invariance and local correlation inherent in convolutional neural networks, the dependence of positional encodings on specific input sizes can be avoided. After feature-map serialization, global constraints were applied to the sequence. When the convolutional layers fail to recognize extremely fine prawn claws owing to their thin structure, the ViT module can utilize the logical context of the entire image to inform the decoder that such features are extensions of the prawn body and must be segmented. The core of the ViT module lies in its multi-head self-attention mechanism, which is formulated as follows:

(5) $$\mathrm{Attention}\left(Q,K,V\right)=\mathrm{Softmax}\left(\frac{QK^{T}}{\sqrt{d_{k}}}\right)V$$

where *Q*, *K*, and *V* represent the query, key, and value matrices obtained by linearly transforming the input sequence. After the self-attention output, the module employs a two-layer linear transformation MLP for nonlinear enhancement along the channel dimensions.

#### 2.2.4. Atrous Spatial Pyramids Pooling

**Figure 6:**
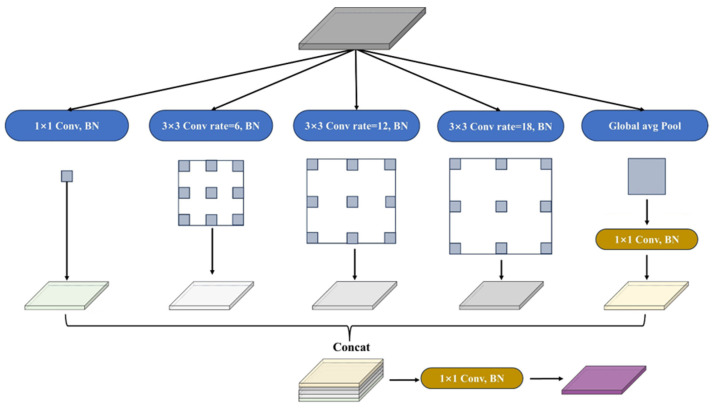
Atrous spatial pyramid pooling module.

One of the primary challenges in the automated segmentation of prawns lies in the scale variability of the target subjects. The body of an adult prawn occupies a relatively large pixel area, whereas the appendages of juvenile or adult prawns, such as slender pereiopods and chelipeds, appear as extremely fine linear structures. To address this, this study introduces an ASPP module at the junction between the encoder and decoder, as illustrated in Figure 6, to achieve the synergistic capture of multi-scale contextual information.

The core of ASPP lies in atrous convolution. Compared to standard convolution, atrous convolution expands the receptive field for feature extraction by introducing a dilation rate d between the elements of the convolutional kernel [19]. The output of the two-dimensional convolution can be expressed as follows:

(6) $$Y\left(i,j\right)=\sum_{m=0}^{M-1}\sum_{n=0}^{N-1}X\left(i+d\cdot m,j+d\cdot n\right)W\left(m,n\right)$$

where *i* and *j* represent the pixel positions in the feature map, m and n represent the indices of the convolution kernel, *M* and *N* indicate the size of the convolution kernel, and d denotes the dilation rate of the atrous convolution. In the implementation of ReViTA-UNet, this study employs three parallel 3 × 3 atrous convolution branches with dilation rates set to *d* ∈ (6, 12, 18). By adjusting the different dilation rates, multi-scale information was captured from the feature map. When *d* = 18 the receptive field was maximized, enabling the extraction of broader biological information across larger spatial spans in the image.

#### 2.2.5. Spatial Attention Upsampling

**Figure 7:**
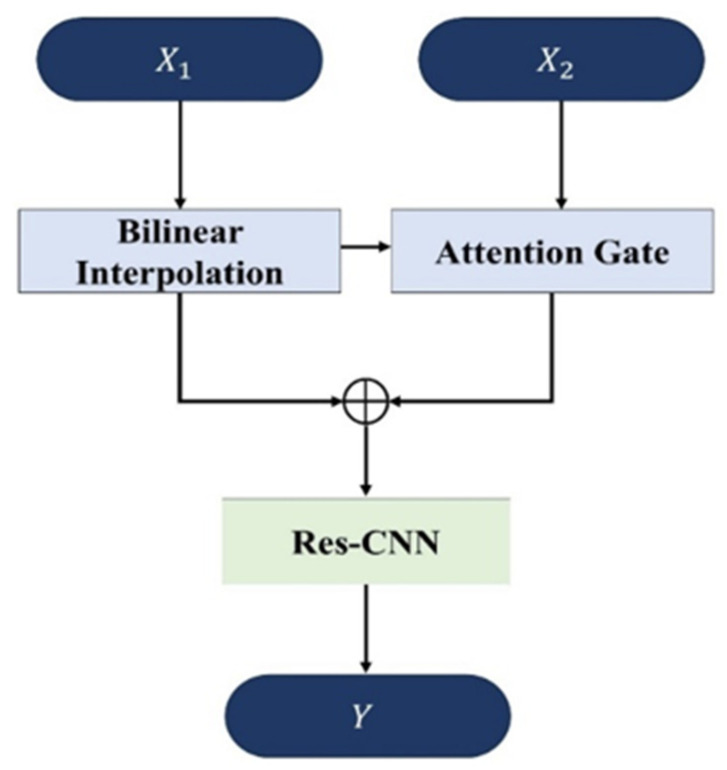
Spatial attention up sampling module.

In the complex image segmentation task for prawns, the traditional U-Net skip connections directly concatenate the shallow features from the encoder with the deep features from the decoder. Although this approach preserves spatial details, it inevitably introduces background noise from shallow layers, often leading to artifacts during prawn segmentation. To address this issue, we enhanced the upsampling module by incorporating an Additive Gating Attention mechanism, as illustrated in Figure 7.

The improved upsampling module first applies bilinear interpolation to upsample the deep features, aligning their resolution with the shallow features from the corresponding encoder layer [20]. Two parallel 1 × 1 convolutional layers then perform element-wise addition on the deep and shallow features to capture the regions of mutual response. The fused features were sequentially processed using a ReLU activation function and a 1 × 1 convolutional projection before being passed through a sigmoid function to generate a spatial attention map. Each value in this map represents the importance of the corresponding pixel, with high-value regions focusing on the prawn body and limb contours and low-value regions corresponding to background noise. Multiplying the original shallow features with the attention map pixel-by-pixel enhances the extraction of useful features, while suppressing interfering information.

#### 2.2.6. Loss Function

In the semantic segmentation task for prawn, the target regions of the prawn body, particularly the slender chelipeds and pereiopods, occupied only a small proportion of the entire image, whereas background regions dominated most of the spatial area. To address this class imbalance problem while maintaining the segmentation performance, this study designed a composite loss function that combines the weighted cross-entropy loss *L_CE_* and Dice loss *L_Dice_* [21]. The cross-entropy loss optimizes the model by measuring the discrepancy between the predicted probability distribution and ground truth annotations. To tackle the issue of sample imbalance, this study introduces a class weight factor λ. The formula used is as follows:

(7) $$L_{CE}=-\frac{1}{N}\sum_{i=1}^{N}\sum_{c=1}^{C}w_{c}\cdot y_{i,c}\log\left(\hat{y_{i,c}}\right)$$

where *N* represents the total number of pixels, *C* denotes the number of classes, *y_i,c_* indicates the ground truth label of the *i*-th pixel belonging to class *c*, and $\hat{y_{i,c}}$ represents the predicted probability of class *c* at the *i*-th pixel.

(8) $$L_{Dice}=1-\frac{2\sum_{i=1}^{N}\hat{y_{i}}y_{i}+\epsilon }{\sum_{i=1}^{N}\hat{y_{i}}+\sum_{i=1}^{N}y_{i}+\epsilon }$$

where *ϵ* is a smoothing factor, set to *ϵ* = 1^−6^, to prevent division by zero.

(9) $$L_{total}=L_{CE}+\lambda \cdot L_{Dice}$$

Here, the optimal value for λ was determined to be 3. This was determined through systematic sensitivity analysis on the validation set. We evaluated λ values ranging from 1.0 to 7.0, and selected λ = 3.0 as it achieved the highest validation performance (Dice: 97.7%, mIoU: 96.5%) with stable training and optimal recovery of fine structures. Given that the prawn body is slender, Dice loss is more sensitive to subtle shape variations. Increasing its weight helps prevent the model from losing fine structures such as pereiopods or chelipeds. However, an excessively high value could lead to overemphasis of the boundary information, resulting in substantial gradient fluctuations during training and hindering the model’s convergence.

### 2.3. Evaluation Metrics

To evaluate the segmentation performance of the proposed model, four standard metrics were employed: Precision, Recall, mean Intersection over Union (mIoU), and Dice coefficient. Precision and Recall measure the accuracy and completeness of pixel-wise classification, respectively. These are complemented by mIoU and Dice, which quantify the spatial overlap between the predicted segmentation masks and the ground truth. Taken together, these indicators provide a comprehensive and balanced assessment of the capability of a model to accurately delineate targets in unstructured environments.

(10) $$Precision=\frac{TP}{TP+FP}$$

(11) $$Recall=\frac{TP}{TP+FN}$$

(12) $$IoU=\frac{TP}{TP+FP+FN}$$

(13) $$mIoU=\frac{1}{n}\sum_{i=0}^{n}{IoU}_{i}$$

(14) $$Dice=\frac{2\times TP}{\left(TP+FN\right)+(TP+FP)}$$

where *TP* (True Positive) represents pixels correctly classified as the target class, *FP* (False Positive) represents pixels incorrectly classified as the target class (background misclassified as foreground), and *FN* (False Negative) represents pixels of the target class that were missed (foreground misclassified as background).

### 2.4. Automatic Analysis System

Based on ReViTA-Unet, we further developed an end-to-end automated measurement system that uses pixel-wise masks generated by the segmentation network as input and integrates geometric topological repair and morphometric parameter computation to acquire biological traits. A geometric repair algorithm was developed to address the fragmentation and discontinuity issues of complex objects. Morphological thinning operators were first applied to extract single-pixel-width skeletons for each functional region. Subsequently, a heuristic reconnection mechanism based on directional coherence restores the continuity between the broken skeleton segments, thereby ensuring that the naturally connected structures remain intact. For extremely fine structures such as cheliped tips, where isolated “pixel islands” frequently occur, a projection-based compensation model indirectly incorporates the geometric contribution of these fragments into the total length, preserving biological information while avoiding the introduction of noisy connections. For compact regions, including the carapace and abdomen, a constrained Voronoi diagram-based medial axis extraction method was employed to generate smooth centerlines that were strictly confined within the contour boundaries, effectively eliminating spurious branches caused by edge irregularities. These components are further discussed in the following sections.

#### 2.4.1. Central Axis Extraction Based on Skeletonization

Conventional region-growing or contour-based algorithms often fail to yield continuous and accurate length measurements of anatomical structures with extremely high aspect ratios such as chelipeds and mouthparts. In real-world farming environments, fine structures may appear fragmented in the segmentation masks because of sensor noise and limited pixel width. However, direct pixel counting introduces a substantial negative bias. To address this, we developed a composite repair algorithm that integrates morphological thinning, geometric potential matching, and linear projection compensation.

(1) Topological refinement and noise pruning: The algorithm performs morphological thinning on the obtained binary mask B ∈ {0, 1}^H×W^. Using a robust parallel thinning operator that preserves topological connectivity, the algorithm compresses the region to a single-pixel-wide centerline. This step effectively eliminates the influence of edge roughness on the subsequent length estimation. To further improve data quality, a branch-pruning strategy based on local operators is introduced. A 3 × 3 convolution kernel was employed for point neighborhood analysis to identify and remove pseudo-branches caused by segmentation artifacts. For each isolated skeleton segment, principal component analysis (PCA) is employed to compute the local tangent vector $\vec{v}$, which served as the initial state for subsequent geometric matching [22].
(2) Heuristic reconnection of broken segments using directional coherence: The segmentation of a single object often yields multiple disconnected skeletal fragments. Therefore, a heuristic reconnection mechanism based on directional coherence is employed. For any two isolated skeleton segments, S_m_ and S_n_, the algorithm first searches the endpoint sets. Let P_m_ be an endpoint of S_m_ and P_n_ an endpoint of S_n_. Define the connection vector as d = P_n_ − P_m_. The merging logic must simultaneously satisfy both the spatial distance threshold and the angular consistency constraint:
  (15) $$\left\|d\right\|<T_{dist}$$

**Figure 8:**
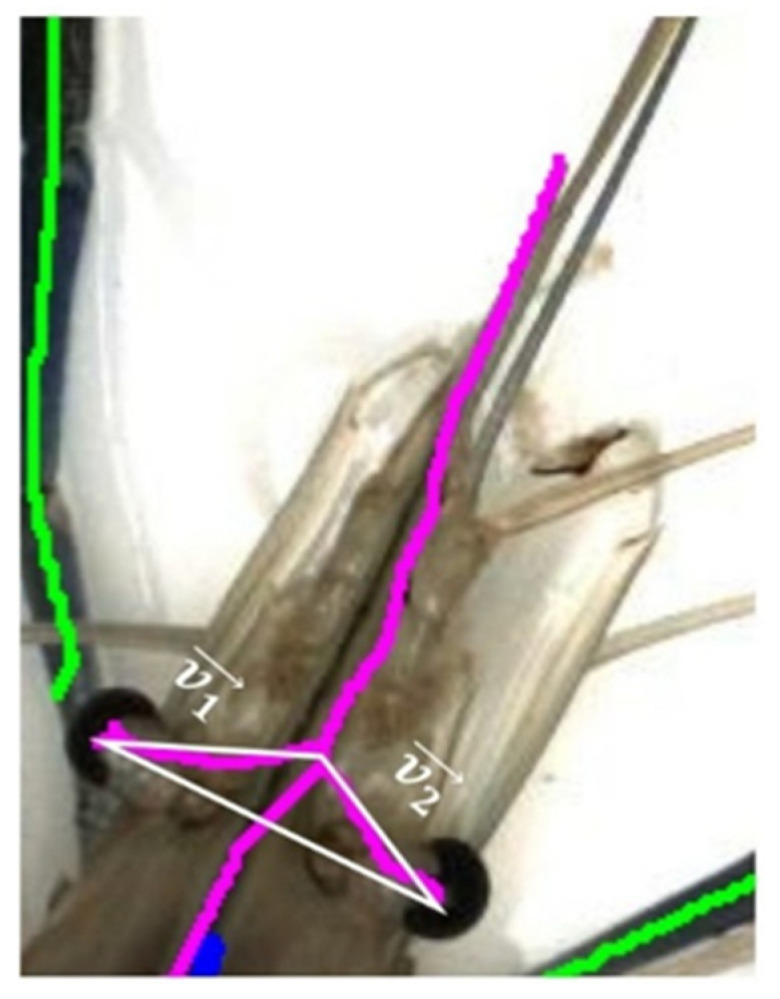
A heuristic reconnection mechanism based on directional coherence for breakpoints.

This formula ensures that two segments possess the possibility of connectivity in the physical space. The mechanism is illustrated in Figure 8.

(16) $$\cos\left(\theta \right)=\frac{\vec{v_{1}}\cdot \vec{v_{2}}}{\left|\vec{v_{1}}||\vec{v_{2}}\right|}<-0.9$$

where $\vec{v_{1}}$ is the tangent vector at P_m_ pointing to S_m_ and $\vec{v_{2}}$ is the vector from P_m_ pointing to P_n_. The cosine constraint enforces near-perfect antiparallelism, that is, an angle close to 180° between the two fragments. The threshold of −0.9 effectively prevents mistakenly connecting two parallel but distinct chelipeds. The distance threshold *T_dist_* and the cosine threshold were both determined through sensitivity analysis on the validation set. *T_dist_* = 20 pixels and the cosine threshold of −0.9 yielded the optimal balance between fragment connectivity and false positive suppression.

**Figure 9:**
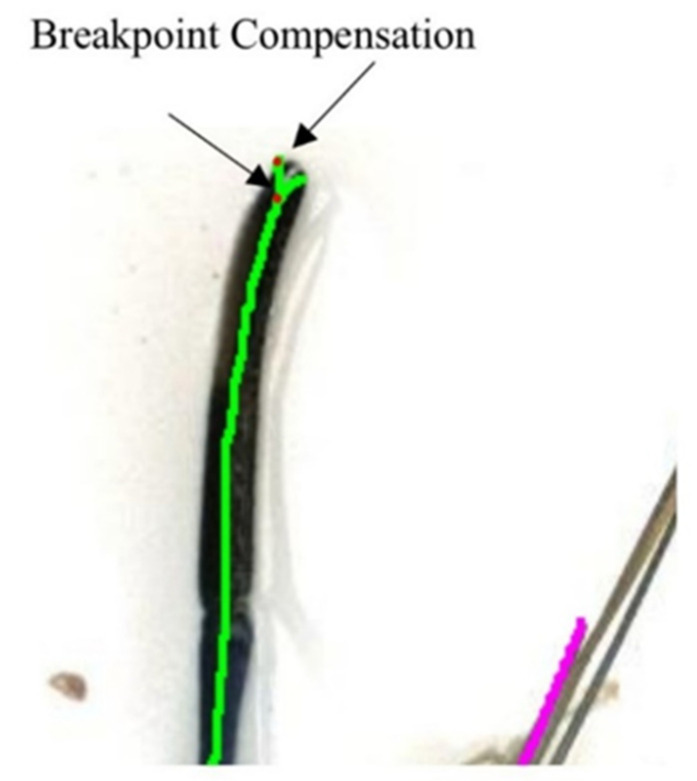
Breakpoint compensation model for fragmented projections.

(3) Compensation model for fragmented projections: In extremely thin structures, such as the cheliped tips, where the pixel width may span only 1–2 pixels, neural networks frequently produce isolated “pixel islands” scattered around the main stem. Ignoring these fragments leads to underestimation of their lengths, whereas indiscriminate connection introduces excessive noise. To address this issue, we designed a geometric projection-based compensation scheme called breakpoint compensation shown in Figure 9.

The algorithm first identifies the two longest connected components as the “main stem.” For each remaining small fragment *D_i_*, the Euclidean distance from its centroid *C_i_* to the endpoint *P_end_* of the main stem is calculated. The missing length attributable to the fragment is estimated by projecting the displacement vector between *C_i_* and the nearest endpoint *P_end_* of the main stem in the local tangent direction as follows:

(17) $$\Delta L=|C_{i}-P_{end}|\cdot |cos(\phi )|+Len(D_{i})$$

where ϕ represents the angle between the centroid-to-endpoint line and the tangent at *P_end_*. This “indirect compensation” approach adds the biological contribution of each fragment to the total length without forcing a noisy direct connection [23], which could significantly improve the robustness of the proposed system against low-quality images.

(4) Path integration and polynomial smoothing: After completing topological repair, local smoothing is applied to the final ordered point set using high-order B-splines or quadratic polynomials to eliminate the staircase effect caused by discrete pixel coordinates [24]. The final length L was obtained by performing line integration along the smoothed parametric curve as follows:
  (18) $$L=\int_{a}^{b}\sqrt{1+{\left[f^{\prime }\left(x\right)\right]}^{2}}dx\approx \sum_{i=1}^{n-1}\sqrt{{\left(x_{i+1}-x_{i}\right)}^{2}+{\left(y_{i+1}-y_{i}\right)}^{2}}$$

This method ensures that, even for highly curved appendages, the measured length closely approximates the true anatomical length.

#### 2.4.2. Medial Axis Extraction via Constrained Voronoi Diagram and Polynomial Regression

**Figure 10:**
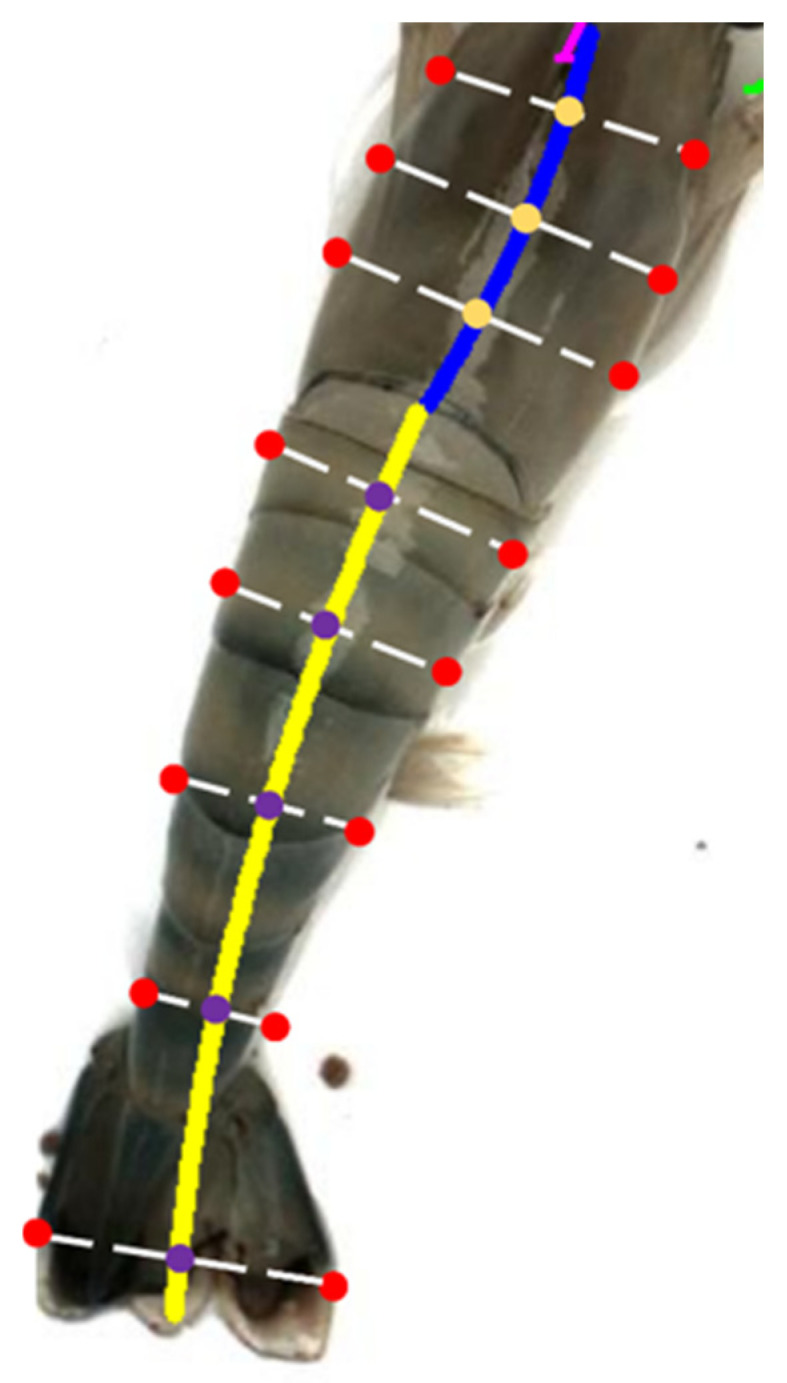
Medial Axis Extraction Based on Constrained Voronoi Diagram.

For compact body parts such as the carapace and abdomen, conventional skeletonization algorithms are prone to edge noise, leading to a “burr phenomenon” that results in overestimation of length calculations. To mitigate this issue and obtain a smooth, anatomically meaningful centerline, we developed a refined central-axis extraction algorithm based on the Voronoi diagram [10] (Figure 10).

(1) Constrained Voronoi Vertex Generation and Filtering: The segmented head and tail contours C of the target region of prawns were sampled at equal intervals to generate a point set P. Based on the medial axis transform theory, local centers equidistant from the boundaries were identified as vertices. Subsequently, a point set P is used to construct a Voronoi space, yielding a set of vertices *V* [25]. Finally, an interior constraint operator was introduced that employs point-in-polygon testing to eliminate redundant vertices located outside the contours, retaining only the core vertex set *V_int_*. The formula used is as follows:
  (19) $$V_{int}=\{v\in V|\mathrm{dist}\left(v,C\right)\geq 0\}$$
(2) Topological Sequence Reconstruction: Since the Voronoi vertex set initially exists as an unordered point cloud, it cannot be directly used for line integration. Instead, PCA was used to determine the primary anatomical extension direction of the object. By computing the covariance matrix of *V_int_* and extracting the eigenvector $\vec{e_{main}}$ corresponding to the largest eigenvalue, all vertices are projected in this principal direction:
  (20) $$\mathrm{proj}\left(v\right)=v\cdot \vec{e_{main}}$$

By sorting the vertices according to their projected scalar values, the disordered point cloud was successfully transformed into a sequence with topological order, effectively resolving the multi-valued problem of bent organisms in the coordinate system [26].

To ensure that the extracted fitted curve remained within the physical boundaries of the organism at both the head and tail, the algorithm performs high-density resampling and dynamic truncation within the parametric space *t* ∈ [−0.1, 1.1]. Only the curved segments that pass the boundary-closure inspection were retained. This approach guarantees global smoothness of the central axis and ensures that the measurement path strictly adheres to the anatomical characteristics of prawn body shape.

(21) $$x\left(t\right)=a_{2}t^{2}+a_{1}t+a_{0},y\left(t\right)=b_{2}t^{2}+b_{1}t+b_{0}$$

(22) $$f\left(t\right)=\left[x\left(t\right),y\left(t\right)\right]$$

where *f*(*t*) represents the fitted two-dimensional parametric vector function defining the continuous trajectory of the prawn’s central axis in the pixel plane; t denotes the normalized parameter; *x*(*t*) and *y*(*t*) indicate the coordinates of the central axis in the segmented image; and a, b represent the undetermined coefficients in the *x* and *y* directions, respectively. To quantify the contribution of each geometric repair stage, we performed a component-wise ablation study on 100 random test images. The complete pipeline reduced body length MAPE from 7.83% (raw pixel counting) to 1.83%, a 76.6% reduction, with skeletonization, directional reconnection, and projection compensation each contributing substantially to this improvement. This confirms that measurement accuracy depends critically on geometric post-processing, not solely on segmentation quality.

## 3. Results and Discussion

### 3.1. Ablation Studies

A series of ablation experiments was conducted to quantify the contribution of individual architectural components to overall segmentation performance in ReViTA-UNet. The original U-Net was used as the baseline. Five model variants (Models A–E) were constructed by sequentially embedding the key modules, including Res-CNN, ViT, ASPP, and the attention-based up-sampling mechanism. Incremental performance gains were evaluated on a consistent dataset to ensure comparability across configurations. The experimental results, evaluated in terms of Precision, Recall, and Dice coefficient, are presented in Table 1.

Baseline U-Net (Model A) achieved a precision of 93.8 ± 0.9%, a recall of 81.6 ± 1.4%, an mIoU of 79.2 ± 1.3%, and a Dice of 84.0 ± 1.0%. The low recall indicates incomplete recovery of target regions, particularly elongated structures such as chelipeds. This limitation is consistent with observations in agricultural segmentation tasks, where conventional architectures often underperform because of insufficient feature representation under complex field conditions [27]. The integration of the residual convolution modules (Model B) yielded satisfactory improvements across all metrics: precision increased to 96.2 ± 0.5%, recall to 95.8 ± 0.6%, mIoU to 92.6 ± 0.7%, and Dice to 93.9 ± 0.5%. The evident gain in recall (14.2% improvement) suggests that residual connections effectively alleviate the vanishing gradient problem during training, enabling deeper feature extraction and more complete region recovery. This is consistent with the report that residual U-Net architectures have demonstrated superior performance in segmentation under high-density conditions [16].

The addition of the Vision Transformer module (Model C) further refined the performance, achieving a precision of 96.7 ± 0.4%, a recall of 95.7 ± 0.5%, an mIoU of 93.1 ± 0.5%, and a Dice of 95.0 ± 0.4%. The modest improvement in mIoU (0.5%) suggests that the Transformer’s primary benefit lies in boundary precision enhancement rather than regional coverage expansion. This is consistent with the finding that self-attention mechanisms excel at preserving fine-grained spatial details. Recent studies have demonstrated that Vision Transformers excel in preserving fine-grained spatial details through parallel patch processing, making them particularly effective for detecting low-contrast regions [28]. In the context of prawn segmentation, this translates into a more accurate delineation of subtle anatomical features, such as antennal tips and telson boundaries. Incorporating the ASPP module (Model D) produced balanced improvements, with the precision reaching 97.0 ± 0.3%, recall 97.2 ± 0.3%, mIoU 94.8 ± 0.4%, and Dice 96.3 ± 0.3%. The concurrent increase in precision and recall indicates that ASPP effectively captures the multi-scale characteristics of prawn morphology. The ViT’s global attention mechanism enhances large-object segmentation but may slightly compromise fine structures such as cheliped tips and rostrum, a phenomenon we term the “global smoothing effect.” This local–global trade-off is subsequently resolved by the ASPP and attention-up modules, which restore multi-scale contextual information and refine boundary localization [29]. This multi-scale aggregation is particularly valuable for imaging aquatic species, in which varying distances and natural postures introduce significant scale variations. The synergistic combination of both components further addresses issues such as blurred segmentation edges and shape distortion caused by the variable sizes of the target objects.

**Figure 11:**
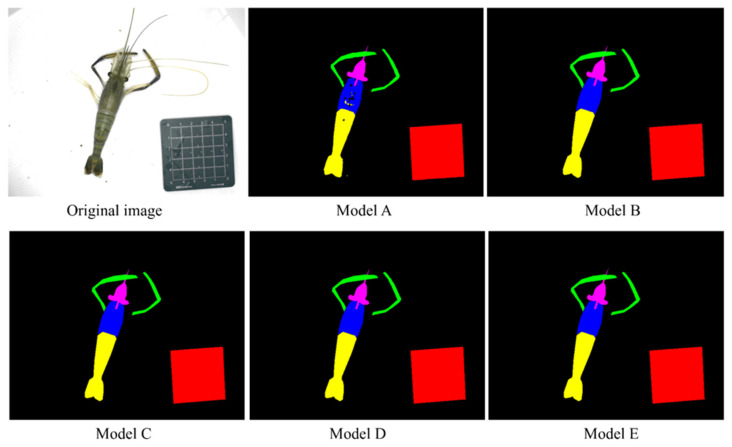
The incremental performance gain of each module in the ablation studies.

Finally, the complete ReViTA-UNet (Model E), which integrates all modules with the attention-up mechanism, achieved the highest performance across all metrics: a precision of 98.3 ± 0.2%, a recall of 98.4 ± 0.2%, a mIoU of 96.7 ± 0.2%, and a Dice of 97.7 ± 0.2%. The attention-guided upsampling module further improved performance, particularly mIoU (1.9% over Model D), by refining decoder feature maps through spatial attention. By adaptively filtering high-frequency details at the pixel level, the module can precisely guide the fusion of deep semantic information with shallow spatial features. In this process, attention-up module effectively suppresses the background noise interference in shallow feature maps and enhances the prediction accuracy of the target boundaries. This enhancement is consistent with the principles of consistency training in semantic segmentation, where perturbations applied to the encoder outputs can improve the representation quality [30]. The progressive improvement from Models A to E demonstrates the complementary nature of the integrated modules. Residual connections provide training stability and depth, transformers contribute to global context and fine detail preservation, ASPP ensures multi-scale feature aggregation, and attention-up refines the boundary localization. Collectively, these components enable robust segmentation performance suitable for downstream morphometric analysis, with strong agreement with ground-truth annotations. The comparative effects of the ablation experiments are shown in Figure 11.

The superior performance of ReViTA-UNet likely reflects its architectural adaptations to prawn morphology, particularly the elongated, high-aspect-ratio structures that challenge conventional architectures. The ablation study confirmed that each architectural component contributed meaningfully to overcoming these domain-specific challenges.

### 3.2. Model Comparison

To assess the robustness and comparative validity of the experimental findings, we benchmarked the proposed model against several mainstream deep-learning segmentation architectures, including the classical U-Net and Fully Convolutional Network (FCN), as well as more advanced networks such as DeepLabV3+, PSPNet, and OCRNet. To ensure rigorous comparison, all models were trained under identical settings: batch size 2, initial learning rate 1 × 10^−4^, Weight Decay 1 × 10^−8^ with AdamW optimizer, 100 epochs with early stopping, and the same data augmentation strategy. Five independent training runs were performed for each model. The results are reported as mean ± standard deviation in Table 2. One-way ANOVA with Tukey’s HSD post hoc test confirmed that ReViTA-UNet significantly outperforms all comparison models across all metrics (*p* < 0.001), with the exception of recall versus ANN (*p* = 0.067). The low standard deviations (<0.5% for ReViTA-UNet) confirm the training stability and reproducibility of our results.

The baseline U-Net architecture achieved the lowest performance among all compared models, with a recall of only 81.6 ± 1.4% and an mIoU of 79.2 ± 1.3% (Table 2). This inferior performance likely reflects the limited capacity of the U-Net to capture long-range dependencies and multi-scale contextual information [31]. This is a critical requirement for the segmentation of elongated structures, such as antennae and chelipeds, that span across substantial portions of the image. The FCN demonstrated strong performance with a precision of 95.3 ± 0.5%, recall of 96.3 ± 0.4%, mIoU of 92.2 ± 0.5%, and Dice of 95.8 ± 0.3%. As a pioneering end-to-end semantic segmentation framework, FCN replaces fully connected layers with convolutional layers to enable dense pixel-wise prediction [38]. However, its fixed receptive field limits its ability to simultaneously capture fine local details and global structural context. PSPNet achieved comparable results (precision 95.4 ± 0.4%, recall 95.4 ± 0.5%, mIoU 91.4 ± 0.7%, and Dice 95.4 ± 0.4%), benefiting from its pyramid pooling module, which aggregates multi-scale contextual information through parallel pooling operations at different grid scales [39]. This multi-scale aggregation is particularly relevant for prawn morphology, in which the model must handle substantial scale variations between the broad thoracic regions and slender appendages. Zhao et al. demonstrated that PSPNet’s global scene prior effectively resolves context mismatches common in complex scene parsing tasks [33]. This principle translates well to biological specimen segmentation, where the anatomical context constrains plausible region boundaries.

DeepLabV3+ exhibits the weakest performance among the compared models. The ASPP module in DeepLabV3+ employs parallel atrous convolutions with different dilation rates to capture features at multiple receptive fields [35]. DeepLabV3+ performed better than PSPNet and other architectures when segmenting fine-grained elongated structures [35]. In addition, poor recall suggests that DeepLabV3+ struggles to completely recover the full extent of prawn appendages, likely owing to insufficient feature preservation during encoding-decoding operations. OCRNet also demonstrated suboptimal performance (mIoU 73.5 ± 1.6%), which was substantially lower than that of other modern architectures. The design of OCRNet focuses on object-contextual representations through pixel-region relations [34], which may be less suited to the elongated, high-aspect-ratio structures characteristic of crustacean morphology, where the regional context differs fundamentally from the compact objects for which OCRNet was originally optimized.

CCNet and GCNet achieved strong and comparable results (mIoU values of 90.0 ± 0.8% and 92.1 ± 0.6%, respectively). Both architectures incorporate sophisticated attention mechanisms: CCNet uses crisscross attention to capture contextual information along horizontal and vertical paths, and GCNet uses simplified nonlocal operations that model the global context. The strong performance of GCNet (95.6 ± 0.4% precision, 95.9 ± 0.5% recall, and 95.8 ± 0.4% Dice) indicates that global context modeling is critical for accurate prawn segmentation, particularly for maintaining coherence across spatially discontinuous yet anatomically connected regions. The ANN achieved the second-best overall performance (mIoU: 92.4 ± 0.6%, Dice: 95.9 ± 0.4%) with a particularly high recall (96.7 ± 0.4%), approaching that of the proposed ReViTA-UNet. This result further suggests that efficient long-range context aggregation can substantially improve segmentation completeness in elongated biological structures.

**Figure 12:**
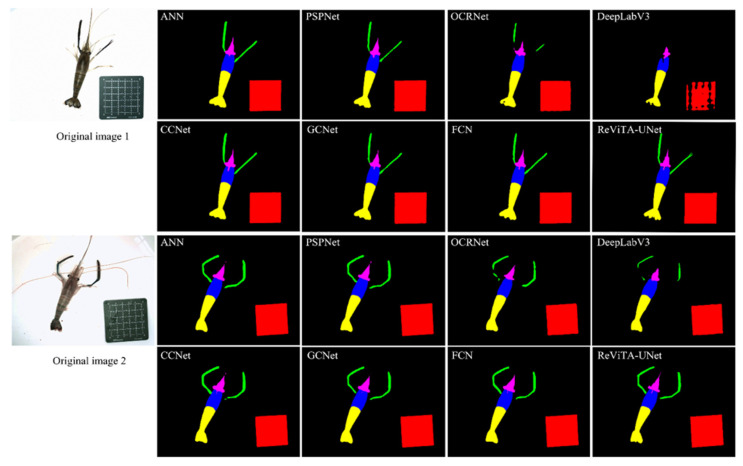
Comparison of segmentation model performance.

The proposed ReViTA-UNet consistently outperformed all comparison models across all evaluation metrics, achieving a precision of 98.3 ± 0.2%, recall of 98.4 ± 0.2%, mIoU of 96.7 ± 0.2%, and Dice of 97.7 ± 0.2%. Compared with the second-best model (ANN), ReViTA-UNet improved mIoU by 4.3% and Dice by 1.8%. These findings indicate that the combined use of attention mechanisms and residual structures substantially strengthens discrimination of target regions. The simultaneous improvement in both precision and recall suggests that through the synergistic integration of multiple architectural modules, ReViTA-UNet effectively reduces both over- and under-segmentation errors, leading to higher overall segmentation reliability. Figure 12 provides a visual comparison of segmentation outputs from two representative image.

Overall, these results indicate that the architectural design is well matched to the challenges of crustacean segmentation, including elongated appendages, compact body regions, and subtle anatomical boundaries that demand fine-grained localization. It is noteworthy that the high recall is particularly significant for downstream morphometric analysis, where complete segmentation is more critical than boundary precision alone; even small omissions in appendage segmentation would propagate directly to length measurement errors. The projection-based compensation mechanism further supports measurement completeness by incorporating fragmented pixel islands into the final estimates, but its effectiveness depends on ReViTA-UNet generating sufficiently complete input masks.

### 3.3. Performance of Automated Prawn Analysis System

**Figure 13:**
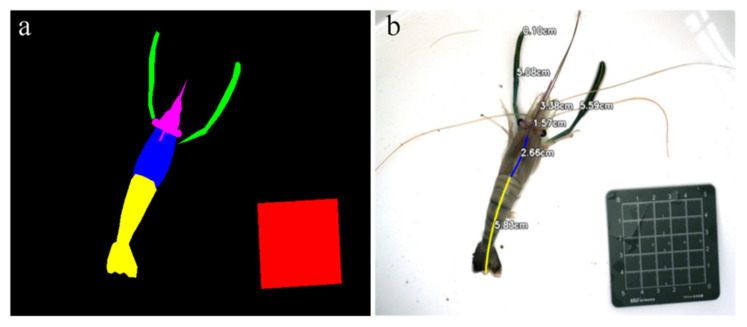
Performance of the auto measurement system. (a) the segmentation result; (b) the auto measurement result.

To evaluate the feasibility of automated measurement of multiple prawn traits using ReViTA-UNet, we developed a corresponding analysis system. The system integrates core functions, such as intelligent segmentation, geometric topology restoration, and parameter calculation, thereby enabling automated conversion of raw input images into biologically quantifiable metrics. The system performance is illustrated in Figure 13.

To assess the practical utility of the proposed system, performance was evaluated using 100 test images. To evaluate the automated measurement system, 100 test images were selected independently from the training data. The selection was stratified to cover the full range of specimen size (15.5–26.3 g), posture (straight, curved, lateral, oblique), and anatomical variation. Quantitative validation was performed by comparing automatically extracted measurements with manual ground-truth measurements from 100 selected images. The mean absolute percentage error (MAPE) and coefficient of determination (R^2^) were calculated for the following four key morphometric traits: body length, carapace length, abdominal length, rostrum length, and eye widths.

As shown in Table 3, the automated system achieved high measurement accuracy for core body traits, with MAPE ranging from 1.83% for body length to 5.24% for cheliped length. Body length exhibited the strongest correlation with manual measurements (R^2^ = 0.987), followed by abdominal (R^2^ = 0.958) and carapace lengths (R^2^ = 0.957). The strong R^2^ values indicate close agreement with manual measurements, and confirm that the system reliably reproduces expert annotations for the principal body regions. The lower accuracies observed for cheliped (MAPE = 5.24%, R^2^ = 0.750) and rostrum (MAPE = 4.87%, R^2^ = 0.817) reflect both algorithmic and reference measurement challenges. First, these structures pose inherent difficulty for the segmentation model, because their extreme aspect ratios and fine distal tips make precise boundary delineation challenging even under optimal imaging. Second, the reference manual measurements could be subject to substantial inter-observer variability. According to our records, independent measurement of 30 specimens by three experts yielded coefficients of variation of 8.7% for cheliped length and 6.2% for rostrum length, compared to only 2.3% for body length. This indicates that it may partly reflect inconsistencies in the ground-truth annotations. Third, cheliped dimorphism introduces protocol bias, because manual measurements may preferentially select the larger cheliped, whereas our system measures the visible cheliped. Fourth, the rostrum’s serrated morphology and ambiguous terminal point create measurement endpoint uncertainty, with manual variation averaging ±0.15 cm. Collectively, these factors explain the lower agreement for these traits. We acknowledge this as an area requiring further investigation and suggest that future studies employ 3D imaging or structured light systems to provide more reliable reference measurements for complex appendages.

Because few studies have examined automated morphometric analysis in *M. rosenbergii*, we compared our system with those reported for related aquatic species. The achieved MAPE of 1.83% for body length compares favorably with that of previously reported automated measurement systems for aquatic organisms. Saleh et al. developed a deep learning-based landmark localization system for black tiger prawn (Penaeus monodon) morphometrics, reporting a MAPE of approximately 2–3% for body length estimation [3]. Although direct numerical comparison is limited by interspecies differences and dataset heterogeneity, the lower error achieved by our system suggests that skeletonization-based approaches may be advantageous for elongated structures, for which landmark interpolation can introduce systematic curvature errors.

## 4. Discussion

In this study, we proposed ReViTA-UNet, an enhanced semantic segmentation model for automated morphometric analysis of *Macrobrachium rosenbergii*, together with a measurement system integrating geometric topological repair and parameter computation. The strong segmentation performance of ReViTA-UNet confirms that combining residual learning with global attention mechanisms is particularly effective for biological specimens with elongated appendages, as residual modules preserve fine structural details while the Vision Transformer compensates for limited CNN receptive fields by establishing long-range dependencies under complex postures. The ASPP module further addresses scale disparity between the compact cephalothorax and slender limbs, while attention-guided up-sampling proves equally critical by suppressing background noise without sacrificing spatial resolution. The notably high Recall (98.4%) is especially meaningful from a practical standpoint, as under-segmentation directly translates into length underestimation that cannot be corrected by post-processing; the competitive Precision (98.3%) confirms that this completeness is not achieved through over-segmentation, striking a balance essential for reliable morphometric analysis. Collectively, these findings suggest that integrating complementary architectural strategies offers a robust pathway for handling the morphological complexity of farmed aquatic species.

Further, the developed auto measuring system performed favorably relative to traditional morphological approaches. Wang et al. [40] applied U-shaped networks combined with anisotropic Gaussian kernel refinement to measure brine shrimp (Artemia) length, achieving a MAPE of 1.16% under controlled laboratory conditions. The slightly higher error observed in our system (1.83%) likely reflects the greater morphological complexity of giant freshwater prawns, which possess multiple articulated appendages and curved body segments absent from the comparatively simple anatomy of brine shrimp. Notably, the carapace length MAPE of 2.14% aligns with established knowledge in crustacean biology. Huang et al. demonstrated that carapace length serves as the most stable predictor for establishing “total length–body weight” regression models in M. rosenbergii [41], but also noted that precise carapace measurement requires accurate identification of multiple anatomical landmarks, such as the rostrum, antennal spines, and branchiostegal spines. The slightly elevated error for carapace length in our system is therefore consistent with this geometric complexity, which challenges both manual and automated measurement.

Although the proposed framework demonstrated excellent performance on the current dataset, several limitations should be acknowledged and addressed in future research.

First, the improved segmentation capability of ReViTA-UNet is achieved at the cost of increased architectural complexity and computational demand. The integration of the Vision Transformer, ASPP, and attention-guided upsampling modules resulted in an average inference time of 172 ms per image on the experimental platform (NVIDIA RTX 4060 GPU). Although this computational overhead may limit deployment on resource-constrained edge devices, the current processing speed is sufficient for laboratory-based phenotyping, selective breeding programs, and other offline analysis scenarios where measurement accuracy and reproducibility are prioritized. In addition, the proposed system provides practical advantages, including non-contact measurement, reduced handling stress, high-throughput processing, and standardized morphometric analysis. Future studies will investigate model optimization strategies, including network pruning, quantization, and knowledge distillation, to achieve a better balance between accuracy and computational efficiency for real-world aquaculture applications.

Second, although the annotated dataset is relatively modest for a Transformer-based architecture, this limitation is not uncommon in biological image segmentation studies [9], where pixel-level annotation of multiple anatomical structures is highly labor-intensive and time-consuming. In this study, each image required detailed annotation of six functional regions to support accurate morphometric analysis, substantially increasing the annotation workload. To reduce the risk of overfitting, extensive data augmentation was applied exclusively to the training set, and the model demonstrated stable performance on the independent validation dataset. Nevertheless, larger and more diverse datasets remain essential to further establish the robustness and generalizability of the proposed framework. Future work will include external validation using images collected from different aquaculture facilities and imaging systems, as well as the exploration of semi-supervised learning strategies to exploit large-scale unannotated datasets.

Third, the current validation was conducted under a controlled imaging environment, where individual prawns were captured under uniform illumination and a clean background. This experimental design enabled reliable evaluation of the proposed framework; however, practical aquaculture environments present additional challenges, including water turbidity, light refraction, surface reflections, variable illumination, floating debris, residual feed, and overlapping individuals. These factors may introduce background interference, reduce boundary localization accuracy, and negatively affect the segmentation of slender anatomical structures such as rostrums and chelipeds. Moreover, the current framework was designed for single-specimen analysis and does not explicitly address severe occlusion or interactions among multiple individuals, which are common in intensive farming systems. Therefore, the proposed system should be considered a laboratory-validated framework rather than a fully deployment-ready solution for commercial aquaculture.

Overall, to facilitate future translation toward practical applications, several research directions will be explored. First, larger datasets containing images acquired under diverse farming conditions will be collected, and advanced augmentation strategies, including generative adversarial networks (GANs), will be investigated to simulate variations in water quality and illumination. Second, occlusion-aware segmentation approaches based on temporal information from video sequences will be developed to improve the analysis of overlapping individuals. Third, multi-site validation across different aquaculture facilities and imaging platforms will be conducted to evaluate the robustness and transferability of the system. These efforts will support the transition of the proposed framework from controlled laboratory experiments toward reliable intelligent monitoring and precision aquaculture applications.

## 5. Conclusions

This study presents ReViTA-UNet, an enhanced semantic segmentation model for automated morphometric analysis of *M. rosenbergii*. By integrating residual convolution modules, a Vision Transformer block, atrous spatial pyramid pooling, and attention-guided upsampling, the architecture is specifically designed to overcome elongated appendages, scale variation, and subtle anatomical boundaries. The proposed framework achieved superior performance, with a Dice coefficient of 97.7% and an mIoU of 96.7%, thereby substantially surpassing eight state-of-the-art models, including U-Net, FCN, DeepLabV3+, and PSPNet. On the basis of this segmentation framework, we further developed an end-to-end automated measurement system integrating geometric topological repair and parameter computation modules. The system achieved a mean absolute percentage error of 1.83% for body length with a strong correlation with manual measurements (R^2^ > 0.96). The multi-strategy geometric repair algorithm, which combines directional coherence-based reconnection, projection-based compensation for fragmented structures, and constrained Voronoi diagram-based medial axis extraction, effectively addresses segmentation imperfections in real-world imaging conditions. The automated system provides practical advantages over conventional manual measurements, such as eliminating handling stress, enabling high-throughput data collection, and delivering standardized measurements. The strong performance under controlled laboratory conditions establishes a foundation for future translation to commercial aquaculture environments. However, we acknowledge that additional validation under field conditions including variable water quality, lighting, and population density is necessary before large-scale commercial deployment. Therefore, future work will focus on multiple individual scenarios, temporal growth monitoring, and enhanced robustness through GAN-based data augmentation for diverse water quality conditions, thereby advancing toward a fully automated intelligent monitoring system for precision aquaculture.

## Figures and Tables

**Table 1:** Ablation studies of ReViTA-UNet.

	Unet	Res-CNN	ViT	ASPP	Attention-Up	P	R	MIoU	Dice
Model A	√					93.8 ± 0.9	81.6 ± 1.4	79.2 ± 1.3	84.0 ± 1.0
Model B	√	√				96.2 ± 0.5	95.8 ± 0.6	92.6 ± 0.7	93.9 ± 0.5
Model C	√	√	√			96.7 ± 0.4	95.7 ± 0.5	93.1 ± 0.5	95.0 ± 0.4
Model D	√	√	√	√		97.0 ± 0.3	97.2 ± 0.3	94.8 ± 0.4	96.3 ± 0.3
Model E	√	√	√	√	√	98.3 ± 0.2	98.4 ± 0.2	96.7 ± 0.2	97.7 ± 0.2

Note: Res-CNN, residual convolution module; ViT, vision transformer module; ASPP, atrous spatial pyramid pooling module; P, precision; R, recall.

**Table 2:** Comparison of model performance for morphology evaluation using different models.

Model	Precision	Recall	mIoU	Dice
U-Net [31]	93.8 ± 0.9	81.6 ± 1.4	79.2 ± 1.3	84.0 ± 1.0
ANN [32]	95.3 ± 0.5	96.7 ± 0.4	92.4 ± 0.6	95.9 ± 0.4
PSPnet [33]	95.4 ± 0.4	95.4 ± 0.5	91.4 ± 0.7	95.4 ± 0.4
OCRNet [34]	91.6 ± 1.0	77.4 ± 1.8	73.5 ± 1.6	83.4 ± 1.2
DeepLabV3+ [35]	93.4 ± 0.8	68.9 ± 2.1	65.9 ± 1.9	76.4 ± 1.3
CCNet [36]	94.0 ± 0.7	94.8 ± 0.6	90.0 ± 0.8	94.3 ± 0.5
GCNet [37]	95.6 ± 0.4	95.9 ± 0.5	92.1 ± 0.6	95.8 ± 0.4
FCN [38]	95.3 ± 0.5	96.3 ± 0.4	92.2 ± 0.5	95.8 ± 0.3
ReViTA-UNet	98.3 ± 0.2	98.4 ± 0.2	96.7 ± 0.2	97.7 ± 0.2

**Table 3:** Performance of the automated measurement system for key morphometric traits.

Trait	MAPE%	R^2^	Mean Manual (cm)	Mean Auto (cm)
Body length	1.83	0.987	12.92	12.88
Carapace length	2.14	0.957	6.22	6.2
Abdomen length	1.96	0.958	6.65	6.62
Rostrum length	4.87	0.817	4.28	4.19
Eye width	3.12	0.868	1.46	1.43
Cheliped length	5.24	0.75	7.39	7.25

## Data Availability

The data presented in this study are available upon request to the corresponding author.
